# Live births after oocyte in vitro maturation with a prematuration step in women with polycystic ovary syndrome

**DOI:** 10.1007/s10815-019-01677-6

**Published:** 2020-01-04

**Authors:** Lan N. Vuong, Anh H. Le, Vu N. A. Ho, Toan D. Pham, Flor Sanchez, Sergio Romero, Michel De Vos, Tuong M. Ho, Robert B. Gilchrist, Johan Smitz

**Affiliations:** 1 Department of Obstetrics and Gynecology, University of Medicine and Pharmacy at Ho Chi Minh City, 217 Hong Bang Street, District 5, Ho Chi Minh City, Vietnam; 2 IVFMD, My Duc Hospital, Ho Chi Minh City, Vietnam; 3 HOPE Research Center, Ho Chi Minh City, Vietnam; 4 Follicle Biology Laboratory, UZ Brussel, Vrije Universiteit Brussel, Laarbeeklaan 101, 1090 Brussel, Belgium; 5 Laboratory of Reproductive Biology and Fertility Preservation, Cayetano Heredia University (UPCH), Lima, Peru; 6 Fertility and Research Centre, School of Women’s and Children’s Health, University of New South Wales Sydney, Sydney, NSW Australia

**Keywords:** In vitro fertilization, In vitro maturation, Polycystic ovary syndrome, Oocyte prematuration, C-type natriuretic peptide

## Abstract

**Purpose:**

Standard oocyte in vitro maturation (IVM) usually results in lower pregnancy rates than in vitro fertilization (IVF). IVM preceded by a prematuration step improves the acquisition of oocyte developmental competence and can enhance embryo quality (EQ). This study evaluated the effectiveness of a biphasic culture system incorporating prematuration and IVM steps (CAPA-IVM) versus standard IVM in women with polycystic ovarian morphology (PCOM).

**Methods:**

Eighty women (age < 38 years, ≥ 25 follicles of 2–9 mm in both ovaries, no major uterine abnormalities) were randomized to undergo CAPA-IVM (*n* = 40) or standard IVM (*n* = 40). CAPA-IVM uses two steps: a 24-h prematuration step with C-type natriuretic peptide-supplemented medium, then 30 h of culture in IVM media supplemented with follicle-stimulating hormone and amphiregulin. Standard IVM was performed using routine protocols.

**Results:**

A significantly higher proportion of oocytes reached metaphase II at 30 h after CAPA-IVM versus standard IVM (63.6 vs 49.0; *p* < 0.001) and the number of good quality embryos per cumulus-oocyte complex tended to be higher (18.9 vs 12.7; *p* = 0.11). Clinical pregnancy rate per embryo transfer was 63.2% in the CAPA-IVM versus 38.5% in the standard IVM group (*p* = 0.04). Live birth rate per embryo transfer was not statistically different between the CAPA-IVM and standard IVM groups (50.0 vs 33.3% [*p* = 0.17]). No malformations were reported and birth weight was similar in the two treatment groups.

**Conclusions:**

Use of the CAPA-IVM system significantly improved maturation and clinical pregnancy rates versus standard IVM in patients with PCOM. Furthermore, live births after CAPA-IVM are reported for the first time.

## Background

In vitro maturation (IVM) of oocytes is an assisted reproductive technology that obviates the use of controlled ovarian stimulation (COS) for in vitro fertilization (IVF) through collection of immature cumulus-oocyte complexes at the prophase I stage, which are then matured in vitro until they reach metaphase II stage [1–3].

The omission of COS means that IVM is particularly suited to patients with polycystic ovary syndrome (PCOS), who have highly variable responses to stimulation with gonadotropins and are at increased risk of exaggerated ovarian response, potentially resulting in significant morbidity, including ovarian hyperstimulation syndrome (OHSS) [4] and ovarian torsion. In addition to improved safety, advantages of IVM over COS include a shortened treatment period, lower costs of medication, and increased convenience as a result of the minimal need for cycle monitoring [2].

Although IVM has several advantages over COS, clinical outcomes were initially suboptimal, with live birth rates per cycle below 30% [5, 6]. More recently, live birth rates have improved to 40% or more in centers with IVM expertise [7, 8]. Nevertheless, retrospective studies of IVM versus COS still demonstrate lower chances of a live birth with IVM [8]. In order for a mature oocyte to successfully support fertilization and embryo development, oocyte competence must be achieved, which is normally only acquired in follicles of pre-ovulatory size [9]. This presents a challenge in the case of IVM because cumulus-oocyte complexes (COCs) are typically collected from small antral follicles. In addition, the maturation rate with non-hCG IVM is lower than that with hCG IVM [10].

The need for enhanced competence of IVM oocytes has driven the development of prematuration (pre-IVM) culture systems [11, 12]. The intent of oocyte prematuration culture is to prevent the spontaneous maturation process that occurs in vitro and to maintain cumulus-oocyte gap junctional communication, which should facilitate ongoing acquisition of oocyte developmental competence in vitro and “capacitate” the oocyte (hence the term capacitation prematuration [CAPA]). This is typically achieved by ensuring high cAMP levels in the COC at oocyte collection and in the early phase or first half of the oocyte culture phase (pre-IVM) [11, 13, 14]. Although this is a new concept in human IVM, it is well established and has been widely practiced for decades in domestic animal IVM [13, 15].

The CAPA-IVM system used was originally developed for zero-stimulation IVM in mice [16], with the key components in the pre-IVM phase being C-type natriuretic peptide (CNP), estradiol, insulin, and low-dose FSH. CNP is a potent meiotic inhibitor, which is a ligand for the granulosa/cumulus cell membrane-bound guanylyl cyclase, natriuretic peptide receptor 2 (NPR2) [17]. CNP activation of NPR2 generates cyclic GMP (cGMP) in the cumulus cells, which is a natural antagonist of the oocyte’s phosphodiesterase type 3A (PDE3A), which thereby prevents spontaneous meiotic resumption of oocytes in vivo or in IVM [18]. Animal literature demonstrates that cGMP/cAMP-mediated pre-IVM systems lead to improved oocyte quality, and embryo and pregnancy outcomes [13–16, 19, 20]. The key known mechanisms of action of such pre-IVM systems are to (1) prevent or delay spontaneous meiotic resumption in vitro; (2) preserve oocyte-cumulus gap junction communication; (3) regulate COC glycolysis and oxidative metabolism; (4) enhance intra-oocyte reduced glutathione levels to counter reactive oxygen species; (5) facilitate the ordered cessation of oocyte RNA synthesis prior to meiotic resumption; (6) decrease meiotic asynchrony between oocytes from follicles of differing size/atresia status; and (7) enable development during pre-IVM culture of a functional EGF signaling network in cumulus cells (reviewed; [11]). The latter point is important because it enables the use of amphiregulin in the second IVM phase of CAPA-IVM to induce meiotic maturation (amphiregulin is one of three EGF-like peptides responsible for natural meiotic maturation in vivo) [21]. Hence, CAPA-IVM is an integrated biphasic IVM system consisting of a prematuration phase that capacitates the oocyte for imminent development and an IVM phase that facilitates ligand-dependent, cumulus cell-driven meiotic maturation of the oocyte [16].

The principle of using CNP in pre-IVM to improve competence of human oocytes has been investigated [22, 23], including safety analyses of resultant blastocysts, which showed similar levels of methylation and gene expression to embryos obtained using COS [24]. However, to date, no studies investigating pregnancy outcomes of any form of pre-IVM in humans, including the CAPA-IVM system, have been undertaken. We hypothesized that using the CAPA-IVM system after minimal ovarian stimulation would improve oocyte developmental competence leading to improved pregnancy outcomes. This prospective study evaluated the effectiveness and safety of the CAPA-IVM system versus standard IVM in women with polycystic ovarian morphology (PCOM) or PCOS.

## Methods

### Study design and subjects

This preliminary, prospective, single-center study enrolled patients between March 2017 and October 2018. Women referred to the clinic (My Duc Hospital, Ho Chi Minh City, Vietnam) to receive infertility treatment were invited to participate if they fulfilled the inclusion criteria (age < 38 years, PCOM [defined as a total antral follicle count ≥ 25 in both ovaries], and no major uterine abnormalities). Exclusion criteria were the presence of high grade endometriosis (American Society of Reproductive Medicine grade > 2), and patients with partners who had cryptozoospermia or azoospermia. All participants provided written informed consent.

**Fig. 1:**
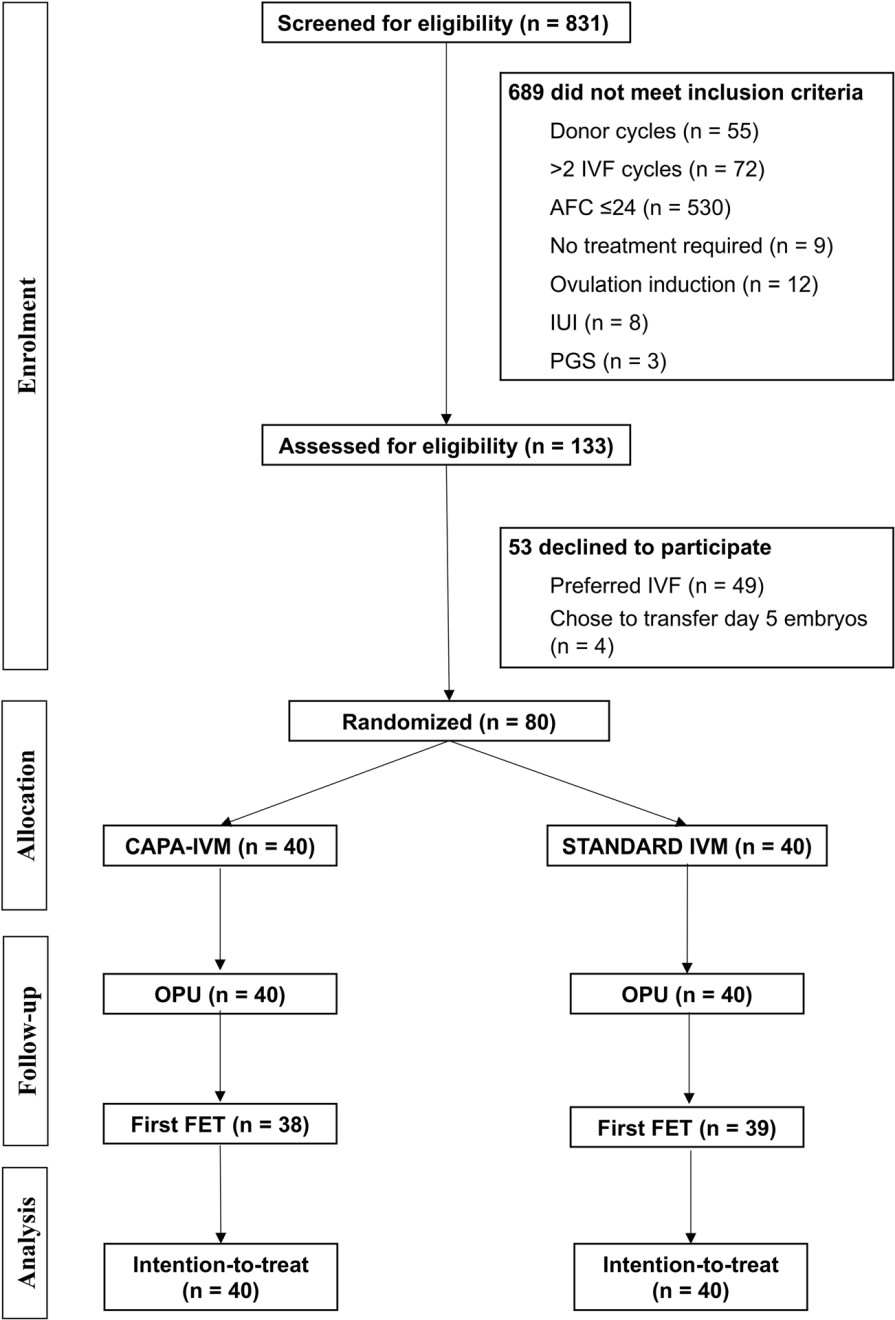
CONSORT diagram. AFC, antral follicle count; CAPA-IVM, capacitation in vitro maturation; FET, frozen embryo transfer; IUI, intrauterine insemination; IVF, in vitro fertilization; IVM, in vitro maturation; OPU, oocyte pick-up; PGS, preimplantation genetic screening

Patients were randomized to receive CAPA-IVM or standard IVM (control) using block randomization by an independent study coordinator using a computer-generated random list (block size 4) (Fig. 1). Due to practical considerations, patients, clinicians, and embryologists were aware of the treatment allocation. Two types of patients with PCOM were allowed to enroll in the study: (1) women with normal menstrual cycle lengths (≤ 35 days), and (2) women with oligomenorrhoea (menstruation occurring at intervals > 35 days with 4–9 periods/year) or total amenorrhea. Diagnosis of PCOS was based on the Rotterdam criteria. Patients were followed up until the end of pregnancy.

The study was performed in accordance with the ICH Harmonised Tripartite Guideline for GCP and the ethical principles of the Declaration of Helsinki. Ethics approval was obtained from the Review Board of the Research Center for Genetics and Reproductive Health and the Ethical Board of My Duc Hospital (approval number 04/17/ĐĐ-BVMĐ, dated 28 March 2017).

### Study schedule and procedures

**Fig. 2:**
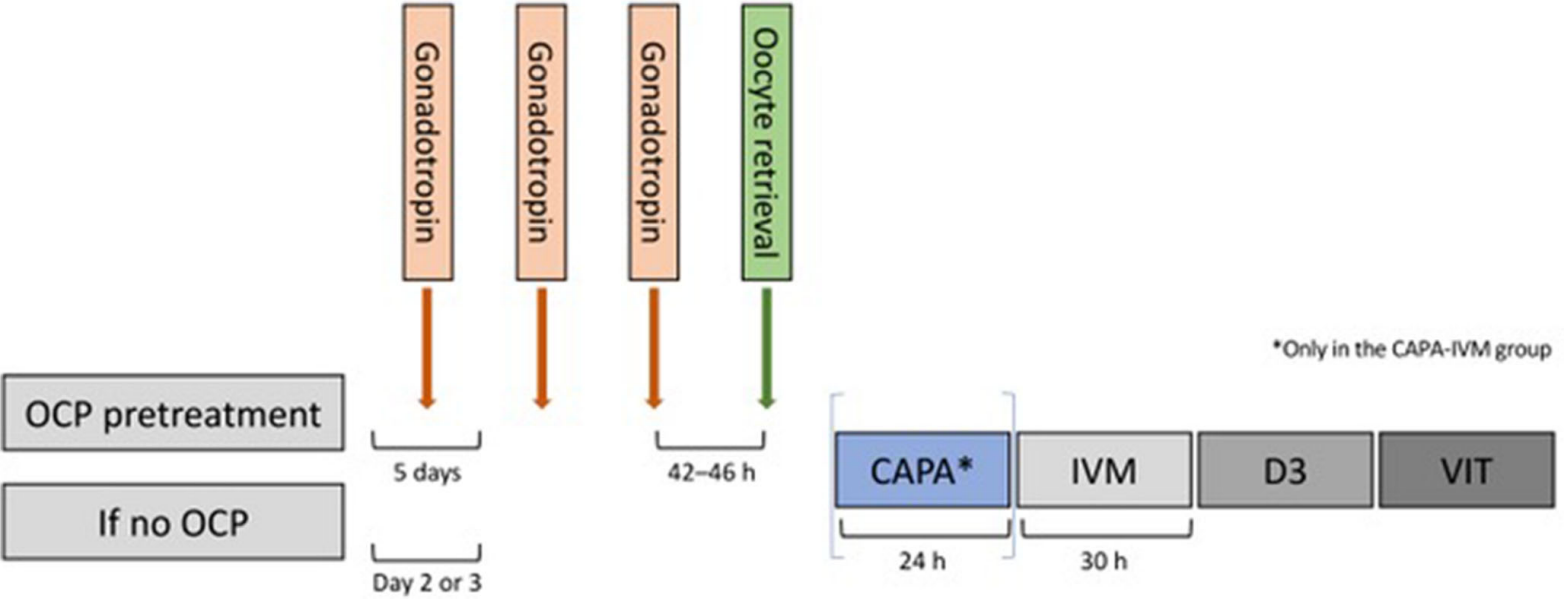
Study schedule for CAPA-IVM and standard IVM (control). Patients were randomized to CAPA-IVM or standard IVM. The stimulation protocols are identical in the two arms. Patients received approximately 2.5 days of gonadotropin priming and no human chorionic gonadotropin (hCG) priming. CAPA-IVM group allocated patients had their oocytes 24-h longer in a prematuration culture (orange bar). All embryos were vitrified and transferred in subsequent cycles. CAPA, capacitation prematuration; D3, day 3; Gn, gonadotrophin; IVM, in vitro maturation; OCP, oral contraceptive pill; VIT, vitrification

At the screening visit, all patients underwent a pelvic ultrasound scan to evaluate their suitability to undergo IVM treatment. Patients with PCOM and normal cycle length (up to 35 days) (group 1) and those with oligomenorrhoea or amenorrhoea (group 2) underwent baseline hormonal profiling that included levels of luteinizing hormone (LH), FSH, 17β-estradiol (E2), progesterone, anti-Müllerian hormone (AMH), sex hormone-binding globulin (SHBG), total testosterone, 17OH-progesterone, prolactin (PRL), thyroid-stimulating hormone (TSH), and anti-thyroperoxidase. Hormonal profiling was included in the study to ensure patients did not have any hormonal disorders (e.g., thyroid conditions), to allow interpretation of any unpredictable outcomes (e.g., no oocytes retrieved, no oocytes maturing after CAPA and/or IVM culture, or no embryo development), and for potential future use as biomarkers to predict outcomes or determine which patients might be the best candidates for CAPA-IVM. In addition, LH, FSH, E2, and progesterone were measured at the first study visit (the day of starting gonadotropin), on the day of the last gonadotropin injection, and at ovum pick-up (OPU). Group 2 patients were required to take 2 weeks of oral contraceptives before the start of the IVM (Fig. 2). Oral contraceptives were given to clear any atretic follicles in the ovary.

Patients in both groups were managed in the same way, except for the 2 weeks of oral contraceptive use in group 2 (Fig. 2). Patients in group 1 had their first clinic visit on day 2 of the menstrual cycle. At this visit, a blood sample was taken for assessment of FSH, LH, estradiol, and progesterone, and they had their first injection of gonadotropin (recombinant follicle-stimulating hormone [rFSH] initially then HP-hMG [Menopur, Ferring] after a change in clinical practice) in the afternoon. In group 2, blood tests and the first gonadotropin injection were introduced on day 5 after discontinuing oral contraceptives, irrespective of whether they had a menstrual period. All patients returned in the morning of the next day (cycle day 3) for ultrasound. If there was a follicle of > 12 mm and all the others were 8–12 mm in diameter (group 1) or there was a follicle ≥ 8 mm in diameter (group 2) then blood hormone levels were determined in the morning and the final dose of gonadotropin was given in the afternoon (2 pm). In both groups, when all follicles were < 8 mm in diameter, another dose of gonadotropin was given, and patients returned the next day for ultrasound, blood tests, and had their final dose of gonadotropin that afternoon (2 pm). The maximum number of gonadotropin injections was three, and OPU was scheduled at 42–46 h after the last gonadotropin injection in all patients. Ultrasound and blood tests to determine FSH, LH, estradiol, and progesterone levels were performed on the day of OPU; hormone levels were determined by electrochemiluminescence on a Cobas Analyser (Roche Diagnostics). OPU was performed by the same two experienced clinicians. During OPU, follicle size was measured before puncture. Larger follicles (≥ 6 mm) were punctured first, then the needle flushed, then smaller follicles (< 6 mm) were punctured. Therefore, each tube contained COCs of a specific size (< 6 or ≥ 6 mm). All supplements were sampled as described previously [22].

Both the control and treatment protocols used for oocyte culture were based on those of Sanchez and colleagues [23]. The control protocol is consistent with normal clinical practice for standard IVM (i.e., non-hCG-primed IVM). Oocytes from patients in the CAPA-IVM group were collected and processed in the presence of CNP as meiotic inhibitor. In the CAPA-IVM group, COCs were plated into a 4-well dish (Nunc, Denmark) at 10 COCs/well using CAPA medium (Medicult IVM medium; Origio, Denmark supplemented with 1 mIU/mL rFSH, 5 ng/mL insulin, 10 nM estradiol, 10 mg/mL human serum albumin [SAGE, Denmark], and 25 nM CNP under oil for 24 h at 37 °C, 6% carbon dioxide in air). After 24 h, COCs were washed and transferred into IVM medium (Origio, Denmark) containing 5 ng/mL insulin, 10 nM estradiol, 100 ng/mL human recombinant amphiregulin, and 100 mIU/mL rFSH, and incubated under oil for 30 h at 37 °C, 6% carbon dioxide in air. Laboratory checks for oocyte maturation were performed after denuding of oocytes at 30 h of IVM for both IVM groups, as previously described [25].

In the standard IVM group (control), COCs were plated into a 4-well dish at 10 COCs/well using IVM medium supplemented with 75 mIU/mL recombinant FSH (Merck, Switzerland), 100 mIU/mL hCG (MSD, USA), 0.01 mg/mL growth hormone (Merck, Switzerland), and 10 mg/mL human serum albumin (SAGE, Denmark). COCs were incubated for 30 h using the same physical and atmospheric conditions as the CAPA-IVM group.

After IVM, matured oocytes were fertilized using intracytoplasmic sperm injection (ICSI) and cultured in an incubator at 37 °C, 5% carbon dioxide, 5% oxygen. Fertilization check was performed at 16–18 h after ICSI. Embryos were cultured to day 3 in Global Total LP (Life Global, Canada) in groups of 2–3 embryos per 30 μL microdroplet. Embryos that fulfilled the freezing criteria were vitrified (Cryotech, Japan) as cleaving day 3 embryos. Embryos of extremely poor quality (Istanbul consensus on embryo quality assessment) defined as fragmentation > 30%, < 6 cells, and multinucleation were not frozen [26]. A freeze-only strategy was used because patients were given gonadotropins for 2–3 days from the second day of their period; therefore, some may still have bleeding and most had a thin endometrium (approximately 5–6 mm).

No fresh embryo transfers were performed. Patients received oral estradiol valerate (Valiera, Laboratorios Recalcine) 2 mg 4 times daily from day 2 of their menstrual cycle. After an estradiol valerate treatment period of at least 10 days and when endometrial thickness was ≥ 8 mm, progesterone (Cyclogest, Actavis) 200 mg was administered intravaginally 4 times daily. Embryo transfer was scheduled 3 days after starting progesterone. Serum beta hCG was tested 14 days after embryo transfer. If a woman became pregnant (beta hCG > 5 mIU/mL), progesterone administration was maintained at the same dose until at least 11 weeks of pregnancy. An ultrasound scan to determine the viability of pregnancy was performed at 7 weeks’ gestation.

### Outcomes

The primary outcome was live birth rate. Secondary outcomes included number of oocytes retrieved; oocyte maturation rate; fertilization rate; numbers of good quality embryos; number of embryos frozen; positive hCG; clinical pregnancy rate; ongoing pregnancy rate; and number of cycles with no oocyte retrieved or no embryos.

### Adverse events

Safety was monitored at each clinic visit or, if any side effects occurred, by questioning and examining the patient, with adverse events and serious adverse events recorded on case report forms. Adverse events were defined as any unexpected medical occurrence (symptoms or signs, abnormal laboratory findings or diseases) that emerged or worsened during the trial, relative to the initial trial visit. Possible adverse events included ectopic pregnancy, miscarriage, medication-related reactions such as overdose, sensitivity and toxicity, and any adverse outcomes related to egg collection. Serious adverse events were defined as any unexpected medical occurrence that resulted in death, was life-threatening, required inpatient hospitalization or prolongation of existing hospitalization, or resulted in persistent or significant disability or incapacitation. Congenital anomaly or birth defect was considered to be serious adverse events.

### Statistical analysis

A key goal of this study was to determine feasibility, acceptability, and outcome variability to aid in planning a larger, adequately powered efficacy trial. Therefore, statistical analyses are primarily descriptive. The planned sample size was 80 patients (40 per group).

Nonparametric statistical methods such as Wilcoxon rank sum were applied to continuous or ordinal outcomes. To estimate 95% confidence intervals (CI) for the difference between two medians, we used bootstrapping and related resampling methods. Outcome rates were estimated for each treatment group, and differences between groups was analyzed using relative risk (RR), 95% CI of RR, and Fisher exact test. Key pregnancy outcomes were analyzed based on intention-to-treat (ITT) basis; secondary analysis per embryo transfer was also performed. A subgroup analysis was performed based on follicular size (< 6 versus ≥ 6 mm). Data are presented as mean values with standard deviation (SD), medians and interquartile ranges (IQRs), or proportions. All analyses were performed using R (Version 3.0.1; R Foundation for Statistical Computing, Vienna, Austria). Statistical significance was defined as *p* < 0.05.

## Results

### Participants

A total of 80 patients were enrolled in the study (40 to each group). There were no significant differences in demographic characteristics, baseline hormone levels, infertility profile, or the proportions of PCO groups 1 and 2 patients randomized to each group (Table 1). Overall, the mean age was 28.3 ± 3.3 years, and mean body mass index (BMI) was 21.6 ± 2.3 kg/m^2^. The mean duration of infertility was 3.0 ± 1.8 years, and most patients had primary infertility. The majority of patients had undergone one previous round of ART, and most had PCOM with oligomenorrhea or amenorrhea (group 2). The gonadotropin used was FSH in the first 40 patients in each group, then there was a change in clinical practice and the second 40 patients in each group received HP-hMG.

**Table 1:** Baseline patient demographics and clinical characteristics

Characteristic	CAPA-IVM (*N* = 40)	Standard IVM (*N* = 40)	*p* value
Age, years	28.5 ± 3.4	28.1 ± 3.1	0.52
Body mass index, kg/m^2^	21.8 ± 2.5	21.4 ± 2.2	0.45
Duration of infertility, years	2.9 ± 1.5	3.0 ± 2.1	0.79
Type of infertility, *n* (%)			0.15
Primary	24 (60.0)	31 (77.5)	
Secondary	16 (40.0)	9 (22.5)	
Number ART attempts, *n* (%)			0.99
1	38 (95.0)	37 (92.5)	
2	2 (5.0)	3 (7.5)	
Diagnosis, *n* (%)			0.35
PCO morphology + normal menstrual cycle length (group 1)	4 (10.0)	8 (20.0)	
PCO morphology + oligomenorrhea/amenorrhea (group 2)	36 (90.0)	32 (80.0)	

Values are mean ± standard deviation, or number of patients (%)

*CAPA* capacitation prematuration, *IVF* in vitro fertilization, *IVM* in vitro maturation, *ART* assisted reproductive technique, *PCO* polycystic ovary, *SD* standard deviation

### Monitoring of ovarian stimulation treatment

**Fig. 3:**
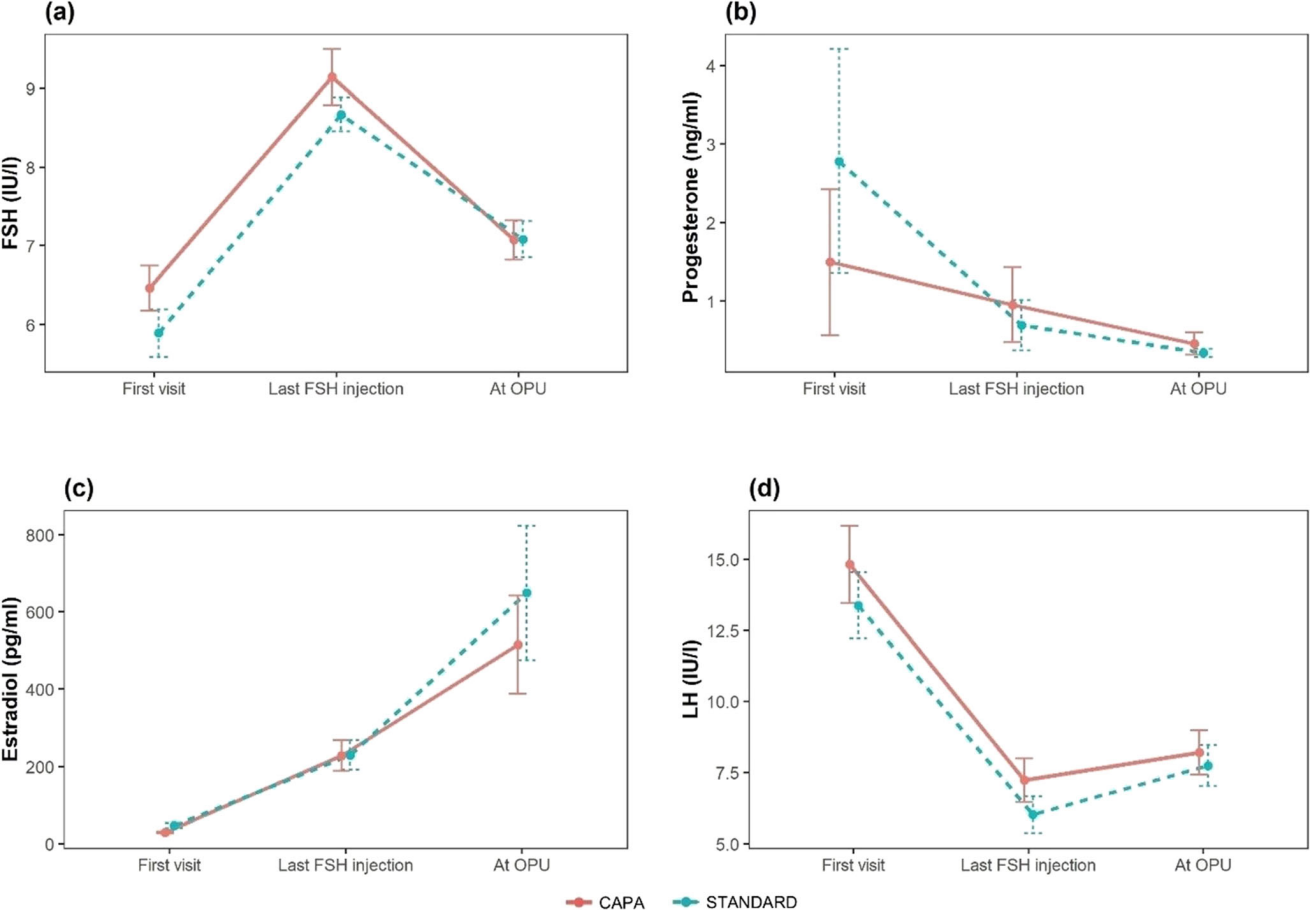
Hormonal profiles during CAPA-IVM (*n* = 40) compared with standard IVM (*n* = 40). Serum concentrations of follicle-stimulating hormone (FSH), luteinizing hormone (LH), estradiol, and progesterone were measured from the day of first visit until the day of immature cumulus-oocyte complex retrieval for both IVM treatments. Values are mean ± standard deviation

The individual monitoring of gonadotrophin, estradiol, and progesterone concentrations over the short treatment period of 4–5 days allowed evaluation of the early follicular response (estradiol increased to > 100 ng/L), absence of increased basal LH concentration and acute LH blips (LH > 10 IU/L), and signs of early luteinization. The FSH and LH profiles were uniform and consistent between treatment groups (Fig. 3).

### Fertility outcomes

The cycle parameters are presented in Table 2. Cycle parameters did not differ significantly between the CAPA-IVM and standard IVM groups, including hormone levels (Fig. 3), the duration of gonadotropin treatment (2.5 days), or the total dose of gonadotropin used (~ 377 IU) (Table 2). Hormonal profiles were also similar in the CAPA-IVM and standard IVM groups (Fig. 3).

**Table 2:** Cycle parameters

Characteristic	CAPA-IVM (*N* = 40)	Standard IVM (*N* = 40)	*p* value
Days on gonadotropins, days	2.5 ± 0.6	2.5 ± 0.5	0.834
Total dose of gonadotropin used, IU	378.8 ± 83.1	375.0 ± 76.0	0.834
Number of follicles at last ultrasound	35.3 ± 15.4	30.9 ± 10.1	0.127
Endometrial thickness on day of OPU (mm)	6.1 ± 2.1	6.1 ± 1.6	0.93

Values are mean ± standard deviation, or number of patients (%)

*CAPA* capacitation prematuration, *FSH* follicle-stimulating hormone, *HP-hMG* highly purified human menotropin, *IVM* in vitro maturation, *LH* luteinizing hormone, *OPU* ovum pick-up

**Fig. 4:**
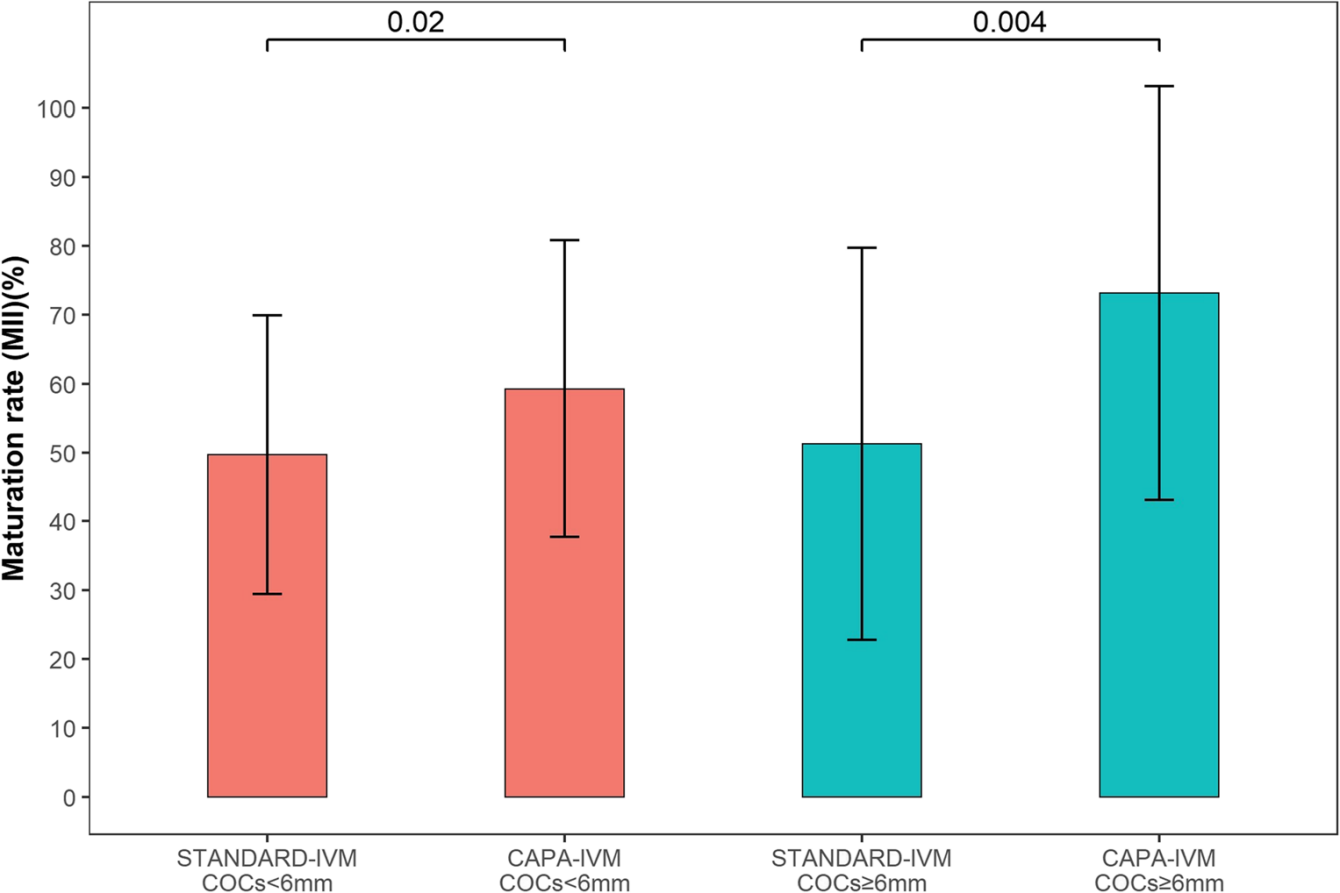
Comparison of maturation rate between standard and CAPA-IVM in follicles of < 6 mm and ≥ 6 mm. Values are mean ± standard deviation; with Tukey’s HSD-adjusted *p* value

After IVM, there was a significantly higher rate of metaphase II oocytes in the CAPA-IVM group versus the standard IVM group (63.6 vs 49.0; *p* < 0.001) and a trend toward an increased number of grade 1 and grade 2 day 3 embryos per COC (18.9 vs 12.7; *p* = 0.11) (Table 3). In the subgroup analysis, improved maturation rates with CAPA-IVM versus standard IVM were observed in oocytes derived from follicles both < 6 and ≥ 6 mm in diameter (Fig. 4).

**Table 3:** In vitro maturation and embryology outcomes

Oocyte developmental outcomes	CAPA-IVM (*N* = 40)	Standard IVM (*N* = 40)	Between-group difference (95% CI)^a^	*p* value^b^
Number of COCs	17.5 [11.0, 23.0]	16.5 [9.8, 21.0]	1 (− 3, 7)	0.39
% Maturation (MII)	63.6 [55.0, 75.0]	49.0 [35.9, 62.1]	14.6 (5.5, 24)	< 0.001
% Pronuclear stage per ICSI	84.0 [72.9, 100.0]	84.50 [72.0, 100.0]	− 0.5 (− 10.7, 11.9)	0.80
% Grade 1 or 2 embryos per pronuclear stage	37.5 [23.8, 50.0]	35.40 [16.4, 50.0]	2.1 (− 11.5, 14.6)	0.60
% Grade 1 or 2 embryos per metaphase II	30.0 [13.9, 43.3]	26.80 [14.3, 40.7]	3.2 (− 8.6, 16.2)	0.70
% Grade 1 or 2 embryos per COC	18.9 [8.5, 26.9]	12.7 [7.3, 20.4]	6.2 (− 1.5, 12.4)	0.11
No embryo, *n* (%)	1 (25)	1 (25)	–	–
Frozen embryos remaining after first ET, *n*	2.5 ± 2.5	1.3 ± 1.9		0.02

Values are median [interquartile range], number of patients (%), mean ± standard deviation, or difference (95% confidence interval)

*CAPA* capacitation culture, *CI* confidence interval, *COC* cumulus-oocyte complex, *EQ1* day 3 embryo quality grade 1, *EQ2* day 3 embryo quality grade 2, *ICSI* intracytoplasmic sperm injection, *IQR* interquartile range, *IVM* in vitro maturation

^a^Bootstrapping and resampling 1000 times

^b^Wilcoxon rank sum test *p* value

The majority of patients had two embryos transferred (Table 4). In the ITT analysis, there was a trend toward a higher implantation rate (*p* = 0.09 vs standard IVM) and higher clinical pregnancy rate in patients in the CAPA-IVM versus standard IVM group (60.0 vs 37.5%; *p* = 0.06), but between-group differences did not reach statistical significance (Table 4). In the secondary analysis by embryo transfer, the implantation rate was also similar between groups (*p* = 0.09) but the between-group difference in clinical pregnancy rate was significantly higher in the CAPA-IVM versus standard IVM group (63.2 vs 38.5%; *p* = 0.04). The rate of ongoing pregnancy was similar between groups (Table 4). There were 19 live births in the CAPA-IVM group and 13 in the standard IVM group (47.5 vs 32.5%, *p* = 0.37; Table 4).

**Table 4:** Pregnancy outcomes after the first transfer

	CAPA-IVM (*N* = 40)	Standard IVM (*N* = 40)	Between-group difference (95% CI)	RR (95% CI)	*p* value
Number of embryos transferred^a^, *n* (%)					0.70
1	3 (7.5)	4 (10.0)			
2	28 (70.0)	31 (77.5)			
3	7 (17.5)	4 (10.0)			
No embryo transfer^a^	2 (5.0)	1 (2.5)			
Pregnancy outcomes^b^					
Positive beta hCG, *n* (%)	24 (60.0)	17 (42.5)	15.2 (− 6.6, 41.6)	1.4 (0.9, 2.2)	0.17
Implantation, *n*	42.1 ± 39.1	26.9 ± 38.0	13.1 (− 2.3, 32.7)	–	0.09
Clinical pregnancy, *n* (%)	24 (60.0)	15 (37.5)	22.5 (− 1.3, 46.3)	1.6 (1, 2.6)	0.06
Miscarriage (before 12 weeks), *n* (%)	4 (10.0)	1 (2.5)	7.5 (− 5.5, 20.5)	4 (0.5, 34.2)	0.39
Ectopic pregnancy, *n* (%)	1 (2.5)	0 (0.0)	–	–	0.63
Ongoing pregnancy, *n* (%)	19 (47.5)	14 (35.0)	12.5 (− 11.4, 36.4)	1.4 (0.8, 2.3)	0.43
Miscarriage (at 12–24 weeks), *n* (%)	0 (0.0)	1 (2.5)	–	–	0.99
Preterm delivery, *n* (%)^c^	2 (5.0)	4 (10.0)	− 5 (− 19, 9)	0.5 (0.1, 2.58)	0.68
< 28 weeks	0 (0.0)	2 (5.0)			–
28 to < 34 weeks	0 (0.0)	0 (0.0)			–
34 to < 37 weeks	2 (5.0)	2 (5.0)			0.99
Gestational age at delivery (weeks)	37.5 ± 1.0	35.1 ± 4.8	2.4 (− 0.6, 5.4)		0.04
Live birth, *n* (%)	19 (47.5)	13 (32.5)	15 (− 8.7, 38.7)	1.5 (0.9, 2.5)	0.37
Singleton	11 (57.9)	8 (61.5)			
Twins	8 (42.1)	5 (38.5)			
Birth weight, g					
Singleton	3045.5 ± 452.5	2691.9 ± 873.4	353.6 (− 401.3, 1108.5)		0.78
Twins	2325.0 ± 450.9	1680.0 ± 639.1	645 (150.6, 1139.4)		0.01

*CAPA* capacitation prematuration, *CI* confidence interval, *hCG* human chorionic gonadotropin, *IVM* in vitro maturation, *RR* risk ratio

^a^One patient has not yet returned for embryo transfer in the CAPA group; two patients had no embryos for transfer (1 in each group)

^b^ Fisher exact test

^c^All preterm newborns were alive in both groups

### Adverse events

In terms of adverse pregnancy outcomes, there were four miscarriages before 12 weeks in the CAPA-IVM group versus one miscarriage in the standard IVM group (10.0 vs 2.5%, *p* = 0.39) (Table 4); no miscarriages occurred from 12 to 24 weeks in the CAPA-IVM group, while one miscarriage occurred in the standard IVM group over this period. One ectopic pregnancy occurred in the CAPA-IVM group (Table 4). No other adverse events were reported. There was no significant difference between groups in singleton birth weight; however, twins were > 0.5 kg heavier in the CAPA-IVM group compared with controls (*p* = 0.01), probably due to the significantly higher gestational age at delivery in the CAPA-IVM group (37.5 vs 35.1 weeks, *p* = 0.04: Table 4). There were no serious adverse events in either treatment arm.

## Discussion

This study is the first to examine pregnancy outcomes in humans after use of an IVM system that incorporates a prematuration step, and is the first to report live births after CAPA-IVM in humans.

In this study comparing CAPA-IVM with standard IVM, a significantly greater proportion of oocytes in the CAPA-IVM group reached metaphase II, showing that oocyte maturation was improved versus standard IVM. Both the implantation rate and the clinical pregnancy rate tended to be higher in patients undergoing CAPA-IVM compared with standard IVM. The live birth rate was not significantly different between the groups. Rates of ectopic pregnancy and miscarriage were low, and although the rate of miscarriage before 12 weeks tended to be higher in the CAPA-IVM, early miscarriage rates were similar to those found in other studies [27, 28]. Although additional research is needed to more reliably determine the miscarriage rate with CAPA-IVM versus standard IVM, the results of the current study support the hypothesis that prematuration IVM systems improve oocyte developmental competence and that this could lead to improved outcomes in human IVM, at least in patients with PCOM like those included in this study.

Consistent with our recent study [24], CAPA-IVM was associated with a significantly higher oocyte maturation rate in follicles < 6 mm in diameter. This improvement in oocyte maturation rate in non-hCG IVF is an important advantage of CAPA-IVM and may have important implications for the efficiency of IVM in the setting of fertility preservation for cancer patients. Indeed, in the latter indication, immature oocytes obtained transvaginally or retrieved from extracorporeal ovarian tissue are mostly derived from small (< 6 mm) antral follicles. The current clinical trial follows our recent preclinical studies [22, 23], which investigated the impact of CNP-mediated IVM (CAPA) on oocyte maturation and embryo yield in humans. Those studies found that there was a significantly greater rate of meiotic maturation of oocytes using CAPA-IVM versus standard IVM (62–70 vs 48%), and an increased yield of good quality embryos per COC [23], similar to the results of the current study.

In the present study, we report a clinical pregnancy rate of 60% using CAPA-IVM compared with 37.5% using standard IVM, and the latter is comparable to rates reported in previous IVM studies [7, 8, 27–30], although an array of different IVM protocols was used in these studies, including some with hCG priming, which was not used in the current study. Clinical pregnancy rates in non-hCG priming frozen embryo transfers after standard IVM were either lower (31.8%) [28] or higher (51.3%) [8] than the 37.5% found in our study. Reported IVM live birth rates range from 24 to 41% [7, 8, 27, 28, 30, 31], similar rates to those found in the present study with standard IVM (37.5%). Nevertheless, live birth rates in studies most similar to ours (no hCG priming and frozen embryo transfer) vary widely, from below (24%) [31] to above (41%) [8]. The standard IVM live birth rate found in our study was 32.5%. In the CAPA-IVM group, the live birth rate was 47.5%, although the difference between the two groups was not statistically significant, this may have been due to the small sample size in this pilot study. Thus, a limitation of this study is the small sample size. Nonetheless, an IVM clinical pregnancy rate of 60% and a live birth rate of 47.5% are favorable outcomes in the broader ART context, and positions this type of IVM as a highly viable method of treating infertility in PCO(S) patients, especially in the context of the many advantages to the patient of IVM over conventional IVF. Another limitation of the study is the transfer of more than one embryo, whereas single embryo transfer should be preferred. The decision to transfer > 1 embryo was because it was thought that IVM embryos were less competent than IVF/ICSI embryos (although this turned out to not be the case). The total number of embryos transferred was 80 in the CAPA-IVM group and 78 in the standard IVM group, but there tended to be slightly more patients with transfer of 3 embryos in the CAPA-IVM versus standard IVM group (*n* = 7 vs *n* = 4 patients; *p* > 0.05), whereas transfer of two embryos was more common in the standard IVM group (*n* = 31 vs *n* = 28 with CAPA-IVM; *p* > 0.05). The rate of twin births in our study was high (approximately 40% overall). This is an indication that IVM embryos have a good capability for implantation. It is also the result of transferring two cleavage embryos, which was standard practice at our center. However, based on the findings of this study, the better option will likely to be move forward to day 5 and transfer of only one blastocyst.

The most important consideration in the development of a novel ART protocol is its safety. The CAPA-IVM system contains a number of novel components not previously used in human ART, although, as detailed in the Introduction, the principles of prematuration IVM systems have been developed from decades of animal research publications and are based on newly recognized natural physiological principles relating to cellular mechanisms regulating oocyte development and maturation (reviewed; [11]). In this context, CAPA-IVM is, in principle, more physiological than standard IVM. The CAPA-IVM media series used in the current study contained no xenobiotic substances; instead the key components (CNP, estradiol, insulin, FSH, amphiregulin) are naturally present in a human follicle (in vivo).

In the first preclinical study of CAPA-IVM using human oocytes [24], all blastocysts of transferable quality on day 5 or 6 were analyzed individually using Next Generation Sequencing (NGS). Blastocysts derived from CAPA-IVM had comparable rates of methylation and expression of major epigenetic regulators to IVF/ICSI blastocysts matched for quality grading and donor age. In the current study, the rate of adverse events (miscarriage, ectopic pregnancy) was comparable between the two study arms and there were no serious adverse events in either group. Gross morphological evaluation of all the babies at birth from CAPA-IVM (*n* = 19) suggests physical normality, similar to findings for the babies born after standard IVM (*n* = 13). As part of this broader clinical research program, detailed molecular analyses of tissue samples from these pregnancies/offspring (including cord blood, placental tissue and buccal smear), as well as detailed follow-up of developmental trajectories of the children, are being undertaken. This pilot study is the first step in the substantial research needed to ensure safety and efficacy of the CAPA-IVM procedure.

## Conclusions

This comparison between CAPA-IVM and standard IVM demonstrates that CAPA-IVM could improve outcomes for women with polycystic ovaries or PCOS seeking treatment for infertility, in terms of significantly increased maturation rates, with potentially better embryo quality and higher pregnancy rates. Further investigations in larger patient populations are warranted in order to determine whether live birth rates with CAPA-IVM are improved compared with standard IVM. To this end, a large randomized clinical trial is currently underway to compare the efficacy and safety of CAPA-IVM versus conventional COS in women with a high antral follicle count (NCT03405701) [25].

## Data Availability

The datasets generated and/or analyzed during the current study are not publicly available due to patient privacy but are available from the corresponding author on reasonable request.
